# Breast cancer prognosis in relation to family history of breast and ovarian cancer

**DOI:** 10.1038/sj.bjc.6601694

**Published:** 2004-02-24

**Authors:** L Thalib, S Wedrén, F Granath, H-O Adami, B Rydh, C Magnusson, P Hall

**Affiliations:** 1 Department of Community Medicine and Behavioural Science (Biostatistics), Faculty of Medicine, Kuwait University, Box 24923, Safat 13110, Kuwait; 2 Department of Medical Epidemiology and Biostatistics, Karolinska Institutet, Box 281, 171 77 Stockholm, Sweden

**Keywords:** breast neoplasms, prognosis, family, registries

## Abstract

We linked four nationwide Swedish population-based registries to identify first-degree family history of breast and ovarian cancer among breast cancer cases diagnosed between 1991 and 1998 and followed them until death, emigration or end of follow-up in December 1998. The median follow-up was 36 months. Using Cox proportional hazards models, the hazard ratio of death (HR) due to breast cancer was estimated. Women with a family history of breast or ovarian cancer (*n*=2175, 12.7%) had a nonsignificantly better prognosis than women without any family history, HR 0.86 (95% CI 0.71–1.05); this appeared unrelated to age at diagnosis either in the index case or in relative(s) with breast and/or ovarian cancer. Our study shows that prognostic outlook is not worse among breast cancer patients with family history.

Family history is a strong risk factor for breast cancer but its prognostic value has not been clearly established. Few population-based studies have addressed this issue and the results are conflicting. Previous investigators have demonstrated family history to be associated with a significantly longer (Fukutomi *et al*, 1993; Malone *et al*, 1996; Mohammed *et al*, 1998; Gajalakshmi *et al*, 1999) or shorter survival (Slattery *et al*, 1993). Some studies found no association between family history and survival (Hermann *et al*, 1985; Anderson and Badzioch, 1986; Israeli *et al*, 1994; Schouten *et al*, 1997; Russo *et al*, 2002). Apart from varying strategies for selection of study population and varying definitions of family history, insufficient sample sizes might explain the differences. We have evaluated the impact of a family history of breast and/or ovarian cancer in first-degree relatives on breast cancer survival by using large Swedish population-based registries.

## PATIENTS AND METHODS

### Data sources

All Swedish residents are assigned an individually unique 10-digit national registration number. This number enables crosslinkage of various nationwide health data registries. Our study links four such registries; The Swedish Cancer Register, The Cause of Death Register, The Multi-Generation Register and The Total Population Register. The Swedish Cancer Register contains information about all cancers diagnosed in Sweden since 1958. It maintains a high degree of completeness due to mandatory reporting by both clinicians and pathologists/cytologists (Mattsson and Wallgren, 1984; National Board of Health and Welfare, 1996). The register does not include information about disease stage or treatment. Established in 1952, the Cause of Death Register provides the date of death, as well as the underlying and contributory causes of death of all deceased Swedish residents. The causes of death are classified according to the *International Statistical Classification of Diseases and Related Health Problems* (ICD), version 6–10. The completeness of the classification of the cause of death in the registry is estimated to exceed 99% (National Board of Health and Welfare, 2001).

The Multi-Generation Register includes all Swedish residents born after 1931, who were alive in 1960, and all those born thereafter. It contains links between children and parents through their national registration numbers. Adoptions and nonbiological relations are flagged. The register is updated yearly. We used the 31 December 2000 version for the present study (Statistics Sweden, 2001:5). The Total Population Register includes information about births and deaths in the entire Swedish population since 1968. The register is updated yearly and kept by the Swedish tax authorities.

The Karolinska Institutet Ethics Committee has reviewed and approved this study.

### Study cohort and family history

In the Swedish Cancer Register, we identified 20 468 women born after 1931, alive in 1960, and diagnosed with primary breast cancer between 1991 and 1998. Our rationale for the choice of this recent study period was that for individuals who died between 1991 and 1998, notification of mothers in the Multi-Generation Register has reached 90% completeness (Statistics Sweden, 2001:5). To obtain follow-up information, we linked the study cohort to the Cause of Death Register and the Total Population Register. The breast cancer cases were followed from the date of breast cancer diagnosis until the end of the study, 31 December 1998, or the date of death or emigration, whichever occurred first. Breast cancer deaths only included those where breast cancer was coded as the underlying cause of death. Linkage of our cohort to the Multi-Generation Register provided us with information about any first-degree relatives (parents, siblings or children) of index cases. We excluded 2729 index patients who were registered as immigrants, and 158 who had been diagnosed with other cancers prior to their breast cancer. We could not identify any relatives for 486 index patients and therefore excluded them. Our cohort thus consisted of 17 095 index breast cancer cases. We identified incident cancers among the relatives of the index cases though rematching with the Swedish Cancer Register. Our inclusion criteria resulted in a maximum attained age at diagnosis that was 59 years in 1991 and 66 years in 1998.

### Statistical methods

We used Cox proportional hazards models to estimate the hazard ratio of death due to breast cancer (HR) and 95% confidence intervals (95% CI) associated with family history. We only considered family history that was apparent at the time of index case diagnosis. Analyses were stratified by age at diagnosis (in 5-year age groups), calendar year (in 5-year intervals) and number of female first-degree relatives in order to control the interdependent effects of treatment time trends, age and varying potential of having a manifested family history of breast and/or ovarian cancer. We estimated survival during the entire follow-up and separately during the first 5 and the following years after diagnosis in order to check the assumption of proportional hazards during follow-up. By introducing interaction terms in the Cox model, we tested if there were differences in the effects of family history on prognosis according to age at diagnosis or follow-up time. All analyses were performed using SAS procedure PROC PHREG (Version 8.2, SAS Institute, NC, USA).

## RESULTS

Out of the 17 095 breast cancer cases we studied, 2175 (about 13%) had at least one first-degree relative with breast and/or ovarian cancer (Table 1

**Table 1:** Various aspects of family history of breast and ovarian cancer among women born after 1931, diagnosed with first breast cancer cases between 1991 and 1998 (*n*=17 095)

	**Number (%)**
*Family history of breast and/or ovarian cancer*	
No	14 920 (87.2%)
Yes	2175 (12.7%)
	
*Number of FDR*^a^ *with breast or ovarian cancer*	
One relative	2090 (12.2 %)
Two or more	85 (0.5 %)
	
*Family history type*	
Breast cancer	1824 (10.7 %)
Ovarian cancer	313 (1.8 %)
Both	38 (0.2 %)
	
*Age at diagnosis in FDR*^a^	
≥50 years	1614 (9.4 %)
<50 years	561 (3.3 %)
	
*Sequence of diagnosis in FDRa relative to index case*	
Before	1883 (11.0 %)
After	292 (1.7 %)

a FDR=first-degree relative(-s).

). In all, 1238 women diagnosed with breast cancer between 1991 and 1998 died of breast cancer, 112 of who had a family history of breast and/or ovarian cancer (Table 2

**Table 2:** Hazard ratios (HRs) for breast cancer death comparing women with and without first-degree family history of breast or ovarian cancer

	**Family history**	**Deaths^a^**	**Person-years**	**HR**	**95% CI**
*Overall*					
	No	1126	50 549	1	Ref
	Yes	112	6039	0.86	0.71–1.05
					
*FDR age at diagnosis*^b^					
<50 years	Yes	43	2299	0.82	0.60–1.12
⩾50 years	Yes	69	3740	0.89	0.70–1.14
					
*Number of FDR with breast or ovarian cancer*^b^					
One	Yes	111	5821	0.89	0.73–1.08
Two or more	Yes	1	218	0.21	0.03–1.49
					
*Stratified by time period after diagnosis*					
First 5 years	No	1061	45 ,115	1	Ref
	Yes	106	5418	0.86	0.70–1.05
					
After first 5 years	No	65	5434	1	Ref
	Yes	6	621	0.94	0.40–2.19
					
*Stratified by index case age at diagnosis*					
<50 years	No	575	23 116	1	Ref
	Yes	64	3054	0.87	0.67–1.13
					
⩾50 years	No	551	27 433	1	Ref
	Yes	48	2985	0.85	0.63–1.15

**Figure 1:**
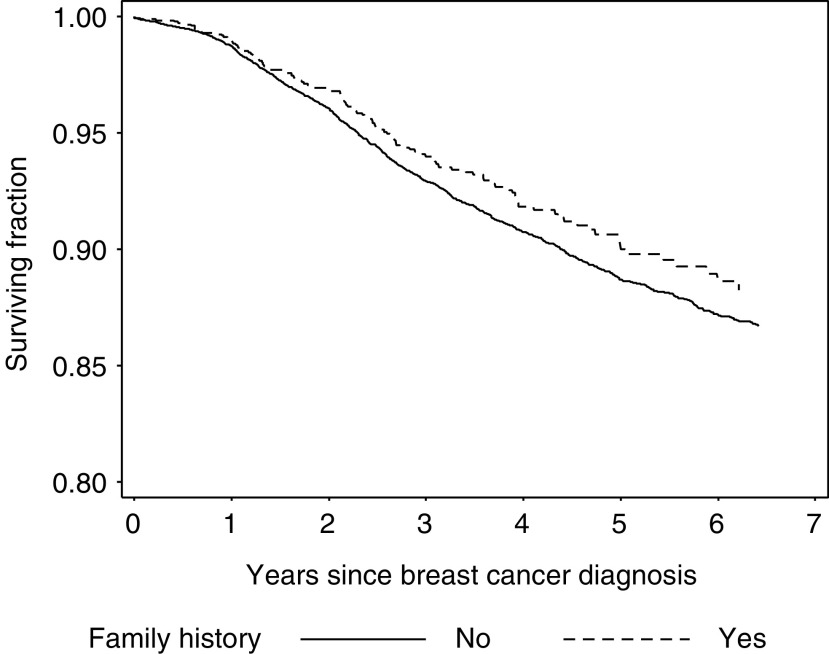
Kaplan–Meier survival curve. Women who died from other causes than breast cancer were censored at the time of death.

95% CI=95 percent confidence interval; HR=hazard ratio; FDR=first-degree relative with breast or ovarian cancer.

a Deaths due to breast cancer.

b Cases with first-degree relatives with breast or ovarian cancer are compared to cases without first-degree relatives with breast or ovarian cancer.

). The mean and median follow-up was 39.6 and 36 months, respectively. Familial cases had a nonsignificantly better prognosis than nonfamilial cases (HR=0.87; 95% CI 0.71–1.05) without any appreciable difference related to duration of follow-up (Table 2, Figure 1

). Among women who had more than one first-degree relative with breast or ovarian cancer, the association seemed particularly pronounced (HR=0.21; 95% CI 0.03–1.53), although this estimate was based on small numbers. The age at diagnosis of the index case or the first-degree relative did not influence the association between family history and breast cancer prognosis (Table 2).

## DISCUSSION

We show that there is no significant difference in the prognosis of breast cancer among women with a first-degree relative of breast or ovarian cancer to cases without family history. The relation was not convincingly modified by duration of follow-up or age at diagnosis in the index case or the relative.

This is one of the largest population-based studies of the relation between family history and breast cancer prognosis published to date. Through linkage of the Multi-Generation and Cancer registries by the national registration numbers instead of relying on reports from the study subjects, we have minimized misclassification of the factor of main interest, family history. Ascertainment of family history was based on the number of first-degree relatives with breast and/or ovarian cancer in the Swedish Cancer Registry. However, due to chance and the high incidence of breast cancer, there may be clustering of sporadic breast cancer in families, giving a spurious impression of familial disease. Indeed, in our data, frequency of family history does not vary substantially over age groups (data not shown). Such clustering would have biased our results towards the null. We did not consider second-degree family history of breast or ovarian cancer. In this regard, Malone *et al* (1996) found that women who had a first-degree family history experienced significantly better prognosis while a positive second-degree family history was not associated with survival.

Given the report of a worse prognosis of familial breast cancer (Slattery *et al*, 1993), our findings are reassuring. However, there may be heterogeneity among familial breast cancers. For example, gene expression pattern has allowed definition of six distinct breast cancer subclasses (Sorlie *et al*, 2001). We could not distinguish such subtypes as we lacked details of gene mutations such as BRCA1 which can influence survival (Stoppa-Lyonnet *et al*, 2000).

We also did not have any information about stage of breast cancer at diagnosis. According to our main hypothesis, breast cancer arising in women with a genetic predisposition might have a different, presumably more aggressive, biologic behaviour than sporadic cancer with a nongenetic aetiology. We pondered whether stage of disease at diagnosis—or any other prognostic factor—could be a confounder when prognosis was compared between familial and sporadic cases. Prevalence of metastases, regional or distant, is the main determinant of stage in a Swedish setting where most cancers are diagnosed with a diameter smaller than 20 mm (Sundquist *et al*, 1999). Since metastatic potential is clearly the main factor in the causal pathway to prognosis, adjustment for this factor in our analysis would be inappropriate. However, delayed clinical diagnosis is another determinant of more advance stage. Confounding would arise if such delay were more or less common in patients with a family history. In this situation, adjustment for stage might eliminate the confounding (provided that stage is accurately classified) but also conceal the key effect we wanted to study. We conclude that adjustment for stage is a questionable approach and that our lack of clinical information is a limited concern.

Previous studies that have compared the survival between breast cancer cases with and without family history found inconsistent results. One reason for the inconsistencies can be varying definitions of family history. An overly generous definition will attenuate any true association between familial disease and prognosis. Where stricter criteria for family history are used, larger proportions of cases will have a genetic component in their aetiology, possibly including specific prognostic implications. Similarly, some investigators identify breast cancer cases in centres for genetic counselling where women with several breast cancer deaths in their families may be over-represented. Other studies have been performed within specific ethnic groups where inherited disease genes are more prevalent, as among Ashkenazi Jews (Robson *et al*, 1999). Another source of variation may be that some studies investigate overall survival that depends heavily on age at diagnosis as well as on socioeconomic factors. Moreover, breast cancer death can occur many years after primary diagnosis and some studies have a short follow-up time. Finally, chance has a role in explaining certain differences from previous studies.

In conclusion, our study shows that prognosis is not worse among familial breast cancer cases overall.
